# Differences in the Endocannabinoid System of Sperm from Fertile and Infertile Men

**DOI:** 10.1371/journal.pone.0047704

**Published:** 2012-10-17

**Authors:** Sheena E. M. Lewis, Cinzia Rapino, Monia Di Tommaso, Mariangela Pucci, Natalia Battista, Rita Paro, Luke Simon, Deborah Lutton, Mauro Maccarrone

**Affiliations:** 1 School of Medicine, Centre for Public Health, Queen’s University Belfast, Institute of Clinical Science, Belfast, United Kingdom; 2 Department of Biomedical Sciences, University of Teramo, Teramo, Italy; 3 Regional Fertility Centre, Royal Jubilee Maternity Services, Belfast, United Kingdom; 4 European Center for Brain Research (CERC)/IRCCS Santa Lucia Foundation, Rome, Italy; University of Rouen, France

## Abstract

Male infertility is a major cause of problems for many couples in conceiving a child. Recently, lifestyle pastimes such as alcohol, tobacco and marijuana have been shown to have further negative effects on male reproduction. The endocannabinoid system (ECS), mainly through the action of anandamide (AEA) and 2-arachidonoylglycerol (2-AG) at cannabinoid (CB_1_, CB_2_) and vanilloid (TRPV1) receptors, plays a crucial role in controlling functionality of sperm, with a clear impact on male reproductive potential. Here, sperm from fertile and infertile men were used to investigate content (through LC-ESI-MS), mRNA (through quantitative RT-PCR), protein (through Western Blotting and ELISA) expression, and functionality (through activity and binding assays) of the main metabolic enzymes of AEA and 2-AG (NAPE-PLD and FAAH, for AEA; DAGL and MAGL for 2-AG), as well as of their binding receptors CB_1_, CB_2_ and TRPV1. Our findings show a marked reduction of AEA and 2-AG content in infertile seminal plasma, paralleled by increased degradation: biosynthesis ratios of both substances in sperm from infertile *versus* fertile men. In addition, TRPV1 binding was detected in fertile sperm but was undetectable in infertile sperm, whereas that of CB_1_ and CB_2_ receptors was not statistically different in the two groups. In conclusion, this study identified unprecedented alterations of the ECS in infertile sperm, that might impact on capacitation and acrosome reaction, and hence fertilization outcomes. These alterations might also point to new biomarkers to determine male reproductive defects, and identify distinct ECS elements as novel targets for therapeutic exploitation of ECS-oriented drugs to treat male fertility problems.

## Introduction

One in six couples has difficulty in conceiving, with the male factor being the primary cause of infertility in 40% of couples. This may depend on a reduced number of sperm due to impaired spermatogenesis or abnormal maturation, or it may be caused by sperm dysfunction from metabolic deregulation or oxidative stress. Recently, lifestyle pastimes such as alcohol, tobacco and marijuana have been shown to have further negative effects on male reproduction [1]–[3].

Conventional semen analysis continues to be the only routine test to diagnose male infertility; however, it cannot discriminate between sperm of fertile and infertile men [4]. For a test to be useful diagnostically or prognostically, it must have little overlap between groups of fertile and infertile men. Routine semen analysis does not meet these standards [4]–[7]. Hence, more sensitive biomarkers of male infertility are urgently needed.

Recent studies [8]–[10] have shown that the endocannabinoid system is a key player in the multifaceted process of male reproduction. In this study we characterised, for the first time, all major components of the ECS in sperm of fertile and infertile men.

Delta-9-tetrahydrocannabinol (THC), the main psychoactive compound extracted from *Cannabis sativa*, impairs spermatogenesis and sperm function, and acts to reduce the release of testosterone [11]–[13]. Anandamide (AEA) and 2-arachidonoylglycerol (2-AG) are the best characterized endocannabinoids (eCBs). Both are endowed with distinct biological activities in the central nervous system (CNS) and in the periphery, where they mimic several actions of THC [14]–[15]. eCBs act principally through cannabinoid receptors, which are members of the rhodopsin family of G protein-coupled seven-transmembrane spanning receptors [16], and include type-1 and type-2 cannabinoid (CB_1_ and CB_2_) receptors as the best characterized targets of eCBs. CB_1_ has been found mainly in the central nervous system [17], but is present also in ovary [18], testis [19], vas deferens [20], and other peripheral endocrine and neurological tissues [21]–[22]. CB_2_ has been found mainly in peripheral and immune cells [23], but also in neuronal cells [24]–[25] and reproductive cells and tissues [26]. Recently, other CB receptors, like the purported “CB_3_” receptor (GPR55) [27]–[28], and non-CB_1_/non-CB_2_ receptors have been identified. Among the latter, non-selective cationic channel type-1 vanilloid receptor (transient receptor potential vanilloid 1, TRPV1), activated by capsaicin and by noxious stimuli like heat and protons, is an additional target of AEA, but not of 2-AG [29]. eCBs are released from membrane phospholipid precursors through the activation of specific phospholipases [30], that are activated “on demand”. Although AEA synthesis may occur via multiple biosynthetic pathways [31], the most prominent route is catalysed by an *N*-acylphosphatidylethanolamine-specific phospholipase D (NAPE-PLD) [32]. Similarly, the formation of 2-AG involves a rapid hydrolysis of inositol phospholipids by a specific phospholipase C (PLC) to generate diacylglycerol (DAG), which is then converted into 2-AG by an *sn*-1-DAG lipase (DAGL) [33]. After re-uptake through a purported specific transporter [34] and intracellular trafficking to selected targets [35]–[36], eCBs signalling is terminated by hydrolysis via fatty acid amide hydrolase (FAAH) [37] for AEA, and via a specific monoacylglycerol lipase (MAGL) for 2-AG [38]. Taken together eCBs, their molecular targets (CB_1_, CB_2_, TRPV1), and their metabolic enzymes form the so-called endocannabinoid system (ECS). Distinct ECS elements have been identified in seminal plasma [39], male reproductive tissues [40], Leydig and Sertoli cells [19], [41]–[42], as well as in male germ cells [41]–[47], from spermatogonia to mature spermatozoa [44], [48]–[50]. Overall, the present evidence supports an “evolutionary” role of ECS (and in particular of CB_1_ and FAAH) as check points in reproduction [3], [9]–[10], [51]–[53].

The presence of *N*-acylethanolamines (NAEs), such as AEA, *N*-palmitoylethanolamine (PEA) and *N*-oleoylethanolamine (OEA), in human seminal plasma [39] further suggests that eCB signalling takes part in regulating capacitation and fertilizing potential within human reproductive tracts. Indeed, evidence for the existence of an active eCBs signalling in sperm has been demonstrated in sea urchin and also in humans [54]–[57]. AEA, through the activation of CB_1_, decreases the motility of human sperm and reduces their capacitation ability [58]. In addition, by activating TRPV1, AEA reduces the fusion of the human sperm membrane with that of the oocyte [48]. However, at present, there are no data on possible alterations of ECS elements in sperm from fertile *versus* infertile men.

The aim of the present study was to investigate the expression and functional activity of the main ECS elements in sperm obtained from fertile and infertile men, in order to ascertain whether alterations in eCBs metabolism and/or receptor activity could be associated with male infertility.

## Materials and Methods

### Reagents

Chemicals were of the purest analytical grade. Anandamide (*N*-arachidonoylethanolamine, AEA) and 5-(1,10-dimethylheptyl)-2-[(1R,5R)-hydroxy-(2R)-(3-hydroxypropyl)-cyclohexyl] phenol (CP55940) were purchased from Sigma Chemical Company (St. Louis, MO, USA). *N*-Arachidonoyl-phosphatidylethanolamine (NArPE) was synthesized from arachidonic acid and phosphatidylethanolamine as reported [59]. [^3^H]CP55,940 (136.9 Ci/mmol), [^3^H]AEA (60 Ci/mmol) and [^3^H]resinferatoxin ([^3^H]RTX, 43 mCi/mmol) were from PerkinElmer Life Sciences (Boston, MA, USA). [^3^H]NArPE (200 Ci/mmol), [^3^H]2-oleoyl-glycerol ([^3^H]2-OG, 20 Ci/mmol) and [^3^H]2-arachidonoylglycerol ([^3^H]2-AG, 200 Ci/mmol) were from American Radiolabeled Chemicals, Inc. (St. Louis, MO, USA). [^14^C]Diacylglycerol ([^14^C]DAG, 56 mCi/mmol) was from Amersham Biosciences. RTX and 2-AG were purchased from Alexis Corporation (San Diego, CA). Deuterated AEA (d_8_-AEA) and 2-AG (d_8_-2-AG) were from Sigma Chemical Company and Cayman Chemicals (Ann Arbor, MI, USA), respectively. Rabbit anti-CB_1_ and anti-MAGL polyclonal antibodies were from Cayman Chemicals; rabbit anti-CB_2_ polyclonal antibody was from Affinity BioReagents (Golden, CO, USA); rabbit anti-NAPE-PLD polyclonal antibody was from Novus Biologicals (Littleton, CO, USA); rabbit anti-FAAH, anti-TRPV1 and anti-β-actin polyclonal antibodies were purchased from Santa Cruz Biotechnology, Inc. (Santa Cruz, CA, USA). Rabbit anti-DAGL polyclonal antibody was from Frontier Science Co. Ltd. (Okkaido, Japan), and horseradish peroxidise (HRP)-conjugated secondary antibody and non-fat dry milk were from Biorad (Hercules, CA, USA). Bovine serum albumin was from Sigma Chemical Company. West Dura Chemiluminescence System and 3,3′,5,5′-tetramethylbenzidine (TMB) were from Pierce (Rockford, IL, USA).

### Semen of Fertile and Infertile Men

This project was approved by the Office for Research Ethics Committees in Northern Ireland and the Royal Group of Hospitals Trust Clinical Governance Committee. The study was conducted at the Regional Fertility Centre, Royal Jubilee Maternity Services, Belfast, Northern Ireland (UK) during the period September, 2005 to December, 2010. Sperm samples for research were obtained after written consent was given by each couple.

Semen from 30 fertile men was obtained from Cryos International, Aarhus (Denmark) and from Androgen Centro Infertilidad Masculina, La Coruna (Spain). Each donor was: a) physically and mentally healthy, b) not suffering from any kind of hereditary disease, c) seronegative for the human immunodeficiency viruses (HIV) 1 and 2, syphilis, viral hepatitis B and C, herpes, cytomegalovirus, d) with no bacterial infection in blood and semen cultures, and e) with a seminal profile exceeding minimal characteristics by WHO guidelines [60].

Semen from 150 infertile men, surplus to clinical requirements, were collected by masturbation after 2–5 days of recommended abstinence.

Following measurement of semen volume, samples were subjected to conventional light microscopic semen analysis within 1 hour of ejaculation, following a period of incubation at 37°C to allow for liquefaction according to WHO recommendations [61], in order to determine sperm concentration and motility. Sperm morphology was assessed according to Kruger Strict Criteria [62]. Following light microscopic analysis, semen was centrifuged at 1500 rpm for 5 minutes. The supernatant was drawn off and the pellet was frozen and stored (−20°C) prior to ECS characterization.

### qRT-PCR Analysis

RNA was extracted from sperm using the RNeasy extraction kit (Qiagen, Crawley, UK), as suggested by the manufacturer. Quantitative real time reverse transcriptase-polymerase chain reaction (qRT-PCR) assays were performed using the SuperScript III Platinum Two-Step qRT-PCR Kit (Invitrogen, Carlsbad, CA, USA) as reported [63]. One µg total RNA was used to produce cDNA with 10 U/µL SuperScript III reverse transcriptase, in the presence of 2 U/µL RNaseOUT, 1.25 µM oligo(dT)20, 1.25 ng/µL random hexamers, 5mM MgCl_2_, 0.5 mM dNTP mix and DEPC-treated water. The reaction was performed using the following qRT-PCR program: 25°C for 10 min, 42°C for 50 min, 85°C for 5 min; then, after addition of 0.1 U/µL of *E. coli* RNase H, the product was incubated at 37°C for 20 min. The target transcripts were amplified using an ABI PRISM 7700 sequence detector system (Applied Biosystems, Foster City, CA), with the following primers: human CB_1_ F (5′-CCTTTTGCTGCCTAAATCCAC-3′); human CB_1_ R (5′-CCACTGCTCAAACATCTGAC-3′); human CB_2_ F (5′-TCAACCCTGTCATCTATGCTC-3′); human CB_2_ R (5′-AGTCAGTCCCAACACTCATC-3′); human TRPV1 F (5′-TCACCTACATCCTCCTGCTC-3′); human TRPV1 R (5′-AAGTTCTTCCAGTGTCTGCC-3′); human NAPE-PLD F (5′-TTGTGAATCCGTGGCCAACATGG-3′); human NAPE-PLD R (5′-TACTGCGATGGTGAAGCACG-3′); human FAAH F (5′-CCCAATGGCTTAAAGGACTG-3′); human FAAH R (5′-ATGAACCGCAGACACAAC-3′); human DAGL F (5′-TTCCAAGGAGTTCGTGACTGC-3′); human DAGL R (5′-TTGAAGGCCTTGTTGTCGCC-3′); human MAGL F (5′-ATGCAGAAAGACTACCCTGGGC-3′); human MAGL R (5′-TTATTCCGAGAGAGCACGC-3′); human β-actin F (5′-TGACCCAGATCATGTTTGAG-3′); human β-actin R (5′-TTAATGTCACGCACGATTTCC-3′). β-Actin was used as housekeeping gene for quantification. One µl of the first strand of cDNA product was used (in triplicate) for amplification in 25 µl reaction solution, containing 12.5 µl of Platinum SYBR Green qPCR SuperMix-UDG (Invitrogen, Carlsbad, CA, USA) and 10 pmol of each primer. The following PCR program was used: 95°C for 10 min; 40 amplification cycles at 95°C for 30 sec, 56°C for 30 sec, and 72°C for 30 sec [63].

### Expression of ECS Elements

Sperm homogenates (50 µg/lane) were subjected to SDS-PAGE on a 10% polyacrylamide gel and electroblotted onto a nitrocellulose membrane as described [63]. Blots were blocked with 10% non-fat dry milk and 5% bovine serum albumin for 2 h, and then incubated with anti-NAPE-PLD (diluted 1∶1000), anti-FAAH (diluted 1∶1000), anti-DAGL (diluted 1∶1000), anti-MAGL (diluted 1∶200), anti-CB_1_ (diluted 1∶100), anti-CB_2_ (diluted 1∶300), anti-TRPV1 (diluted 1∶200) and anti-β-actin (diluted 1∶1000) primary antibodies. After washing, filters were incubated with the horseradish peroxidise (HRP)-conjugated secondary antibody (diluted 1∶1000) and the detection was carried out using West Dura Chemiluminescence System [63]. Protein expression levels were quantified by densitometric analysis, using the ImageJ software after normalization with β-actin [64].

Protein expression of ECS elements was also determined by enzyme linked immunosorbent assay (ELISA), as reported [65]. Briefly, wells were coated with sperm homogenates (20 µg/well) and were incubated for 1 h at room temperature with the same antibodies and at the same dilutions used in Western blotting analysis. After rinsing three times with 5% BSA/PBS-Tween 20, 100 µl of HRP-conjugated secondary antibody (diluted 1∶5000) was added and the ELISA plate was further incubated for 30 min at room temperature. The HRP enzymatic activity was determined by the addition of 100 µL/well of tetramethylbenzidine (TMB) containing H_2_O_2_ (0.002%), and the absorbance was read on a Multiskan ELISA Microplate Reader (Thermo Labsystems, Bevery, MA, USA) at 450 nm.

### AEA Metabolism

The synthesis of [^3^H]AEA by NAPE-PLD was assayed in sperm extracts (200 µg/test), by using 100 µM [^3^H]NArPE and reversed phase-high performance liquid chromatography (RP-HPLC), coupled to online scintillation counting [59]. The hydrolysis of 10 µM [^3^H]AEA by FAAH was assayed in sperm extracts (50 µg/test), by measuring the release of [^3^H]ethanolamine as reported [59].

### 2-AG Metabolism

The synthesis of 2-AG by DAGL was evaluated in sperm homogenates (200 µg/test) by measuring the release of [^14^C]2-AG from [^14^C]DAG by thin layer chromatography and scintillation counting [50]. The hydrolysis of 2-AG by MAGL was assayed by measuring the release of [^3^H]glycerol from [^3^H]2-OG by scintillation counting [50].

### Receptor Binding Assays

For cannabinoid receptors studies, membrane fractions from sperm were prepared as reported [44], and were stored at –80°C. Membrane fractions (50 µg/test) were used in rapid-filtration assays [44] with the synthetic cannabinoid [^3^H]CP55.940 (400 pM), that binds to both CB_1_ and CB_2_ receptors [66].The binding of the TRPV1 agonist [^3^H]RTX (500 pM) was also evaluated by rapid-filtration assays [48]. In all experiments, unspecific binding was determined in the presence of cold agonists (1 µM CP55.940 or 1 µM RTX), as reported [65].

### Endogenous Levels of eCBs

Purified sperm and seminal plasma were subjected to lipid extraction with chloroform/methanol (2∶1, v/v), in the presence of d_8_-AEA and d_8_-2-AG as internal standards [67]. The organic phase was dried and then analysed by liquid chromatography-electrospray ionization-mass spectrometry (LC-ESI-MS), using a single quadrupole API-150X mass spectrometer (Applied Biosystem, CA, USA) coupled with a Perkin Elmer LC system (Perkin Elmer, MA, USA). Quantitative analysis was performed by selected ion recording over the respective sodiated molecular ions [48].

### Statistical Analysis

Data were analyzed in the GraphPad Prism statistical PC program using the non-parametric Mann-Whitney *U*-test (GraphPad Software, San Diego, CA). A level of p<0.05 was considered statistically significant. All data were reported as mean ± S.E.M. of at least three independent experiments, each performed in duplicate.

## Results

### Demographics of Semen from Fertile and Infertile Men

Semen from fertile donors had sperm concentrations ranging from 48–136 million/mL, morphologies of 5–16% and motilities of 24–62% (Table 1). Semen from infertile patients had concentrations of 2–207 million/mL, morphologies of 2–17% and motilities of 1–66% (Table 1). No significant differences were found between fertile and infertile men. These data show that none of the parameters routinely used for semen analysis is indicative of a man’s fertility potential.

**Table 1:** Demographic data of fertile and infertile men.

Parameters	Fertile donors	Infertile men
Men included (n)	30	150
Male age (years)	34.4±0.9	37.6±0.6
Semen volume (ml)	3.2±0.8	3.7±1.3
Sperm concentration(10^6^ml^-1^)	83.3±12.8	68.1±24.7
Progressive motility (%)	45.6±13.9	46.6±14.2
Normal morphology (%)	12.7±3.2	10.1±4.1

Values are expressed as mean ± SD, P>0.005 is NS.

### Expression of ECS Genes and Proteins in Sperm from Fertile and Infertile Men

The results of qRT-PCR experiments on gene expression of the main components of ECS in sperm from fertile and infertile men are shown in Table 2. In terms of AEA metabolism, NAPE-PLD and FAAH genes were expressed to similar extents in both groups. Instead, a significant decrease of DAGL (p<0.01) and MAGL (p<0.05) mRNA levels was found in infertile *versus* fertile sperm. In addition, the mRNA levels of both CB_1_ and CB_2_ receptors were lower in infertile than fertile sperm (p<0.05). Furthermore, a trend towards decreased mRNA levels of TRPV1 was observed in infertile *versus* fertile sperm (Table 2).

**Table 2:** Gene expression at mRNA level of ECS elements in human sperm.

mRNA level^a^	Fertile sperm	Infertile sperm
NAPE-PLD	13.6±8.0	7.0±6.0
FAAH	1.2±0.3	0.4±0.1*
DAGL	25.5±11.7	0.8±0.4**
MAGL	4.1±1.7	0.8±0.5*
CB_1_	57.1±31.6	2.2±1.5*
CB_2_	32.9±23.6	13.5±12.4*
TRPV1	12.9±9.4	7.3±6.2

Values are expressed as mean ± S.E.M.

a Expressed as arbitrary unit. The amount of target transcripts, normalized to the housekeeping gene (β-actin), was calculated using the comparative CT method.

* p<0.05 *versus* fertile.

** p<0.01 *versus* fertile.

**Figure 1:**
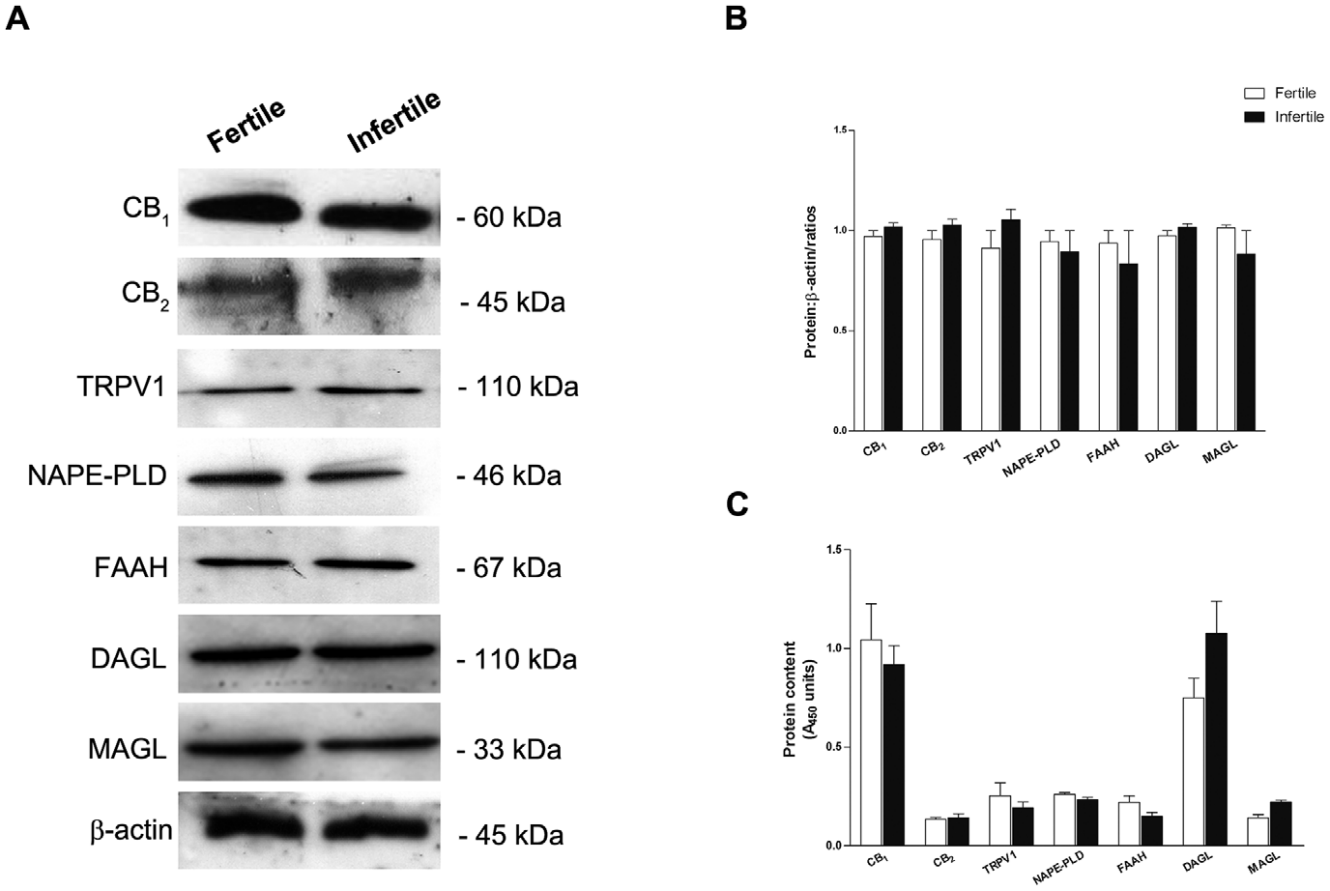
Protein expression of the ECS elements in human sperm. Representative Western blots (A) and densitometric analysis of the immunoreactive bands (B) of the ECS elements in fertile and infertile sperm. The expected molecular mass of each protein is reported on the right-hand side. C) Protein content of the ECS elements determined by ELISA assay in fertile and infertile sperm, expressed as absorbance units at 450 nm.

Next, to determine the possible changes of ECS elements at protein level between fertile and infertile sperm, Western blot analysis was performed. Figure 1A shows a representative immunoblot of fertile *versus* infertile sperm obtained from single donors. Specific anti-NAPE-PLD, anti-FAAH, anti-DAGL, and anti-MAGL antibodies, as well as anti-CB_1_, anti-CB_2_ and anti-TRPV1 antibodies recognized a single immunoreactive band of the expected molecular size, both in fertile and infertile sperm. Protein levels of ECS elements, analyzed by densitometry, did not change between the two groups (Fig. 1B), an observation that was corroborated by a more quantitative ELISA analysis (Fig. 1C). Incidentally, the presence of CB_1_, CB_2_, TRPV1, NAPE-PLD and FAAH in fertile human sperm extends previous findings [48], whereas the presence of DAGL and MAGL in these cells is unprecedented. On a general note, some discrepancies were observed between the mRNA and protein expression of the ECS elements analyzed. However, it should be recalled that disparities among mRNA abundance and protein levels of proteins are not unprecedented [68], also in the context of the ECS [63]–[64], [69]. It can be speculated that distinct regulatory mechanisms of the steady state levels of mRNAs and proteins might be responsible for the observed differences.

### Activity of ECS Elements in Sperm from Fertile and Infertile Men

Table 3 shows the activity of ECS elements tested in sperm from fertile and infertile men. Interestingly, NAPE-PLD and FAAH activities were significantly decreased in sperm from infertile *versus* fertile men. In particular, in infertile sperm NAPE-PLD and FAAH were reduced respectively compared with ∼25% and ∼50% of the values in fertile sperm. In addition, a trend towards decreased DAGL and MAGL activities was observed in infertile *versus* fertile sperm. Interestingly, the ratio between FAAH and NAPE-PLD activity (from ∼19 to ∼40) and that between MAGL and DAGL activity (from ∼2.5 to ∼5.0) almost doubled in infertile *versus* fertile sperm (Table 3). Therefore, infertile sperm seem to improve the overall catabolism of eCBs. Also a slight, yet not statistically significant, decrease of pan-CBR binding was found in sperm from infertile *versus* fertile men, and a dramatic decrease of TRPV1 binding was detected in infertile *versus* fertile sperm. Since the mRNA and protein expression of the latter receptor differed little between fertile and infertile sperm, it is proposed that a different intracellular localization might have masked the binding site of TRPV1 in infertile sperm, thus preventing its accessibility by the ligand. Unfortunately, the paucity of sperm samples from donors with proven fertility did not allow further investigation of this hypothesis, nor was further assessment of receptor functionality possible (*e.g*., in sperm capacitation or calcium mobilization assays). Further studies are needed to elucidate the characteristics of TRPV1 fully.

**Table 3:** Activity of ECS elements in human sperm.

Specific Activity	Fertile sperm	Infertile sperm
NAPE-PLD^a^	57±9	14±2***
FAAH^a^	1067±88	561±155*
DAGL^a^	236±90	140±40
MAGL^a^	620±72	676±44
CBR^b^	147±47	70±21
TRPV1^b^	91±1	< LOD

Values are expressed as mean ± S.E.M.

a Expressed as pmol/min per mg of protein.

b Expressed as fmol/mg of protein.

* p<0.05 *versus* fertile.

*** p<0.0001 *versus* fertile.

LOD, limit of detection (10.0±0.1 fmol/mg of protein).

### Endocannabinoid Levels in Seminal Plasma and Sperm from Fertile and Infertile Men

Consistent with the activity data, a significant reduction in AEA (p<0.0001) and 2-AG (p<0.01) levels was found in seminal plasma of infertile *versus* fertile men, but not in infertile *versus* fertile sperm (Table 4). Moreover, a higher content of 2-AG compared with that of AEA was detected in all groups tested, and overall infertile samples presented a lower amount of eCBs with respect to fertile samples (Table 4).

**Table 4:** Endocannabinoid levels in human sperm.

Endogenous content	Fertile sperm	Infertile sperm
AEA in sperm^a^	0.9±0.3	0.8±0.1
AEA in seminal plasma^b^	26.4±3.6	7.3±1.2***
2-AG in sperm^a^	37.9±9.2	31.3±6.8
2-AG in seminal plasma^b^	218.8±55.4	56.7±14.1**

Values are expressed as mean ± S.E.M.

a Expressed as pmol/mg of protein.

b Expressed as pmol/ml.

** p<0.01 *versus* fertile.

*** p<0.0001 *versus* fertile.

## Discussion

Previous studies demonstrated the presence of a fully functional AEA-related ECS in sperm obtained from sea urchin [43], frog [70], mouse [50], boar [44], bovine [71], and human [48]. Recently, our group has also provided evidence that the AEA-binding TRPV1 receptor could play a role in the acquisition of sperm fertilizing ability in humans [48].

In order to further our understanding of the role of ECS in male fertility, here we investigated AEA and 2-AG metabolism in sperm from infertile *versus* fertile men, aiming at ascertaining any difference in the expression and/or activity of ECS elements possibly associated with male infertility. We found a substantial modulation of AEA metabolism in sperm from infertile men. The biosynthesis of AEA through NAPE-PLD and, to a lesser extent, its degradation by FAAH, were both significantly impaired in infertile *versus* fertile sperm, leading to a significant reduction of AEA content in seminal plasma of infertile sperm. These results are somewhat reminiscent of previous data obtained in maternal lymphocytes, where an association between decreased activity of FAAH and early pregnancy failure was demonstrated [72]; yet, in women who miscarried the AEA content in blood increased [73]. Here, low AEA levels is in keeping with the decreased CB_1_ and CB_2_ binding observed in sperm from infertile men. In this context, high intracellular levels of AEA are essential to promote the fertilizing ability of both boar [44], bovine [74] and human sperm [48], by activating TRPV1 receptors at an intracellular binding site. Indeed, TRPV1 ion channels are key players in capacitation and acrosome reaction [75], which are critical steps in sperm fertilizing ability [76]–[78].

Mammalian sperm cannot penetrate the oocyte’s zona pellucida immediately after ejaculation. A final stage of maturation called capacitation must first be completed. Capacitation is the process during which sperm’s motility pattern changes from progressive motility to a very energetic, non-progressive pattern hyperactivated motility where increased flagellar curvature and wider lateral head movements provide the sperm with more strength to penetrate the outer vestments and cumulus cells of the oocyte. This process is facilitated by a calcium influx. Another feature of capacitation that further aids the process of fertilization is the sperm’s ability to undergo the acrosome reaction. The regulation of this capacitated state is closely associated with the sperm’s proximity to the oocyte. If the process is initiated too early, that sperm will be infertile [76]–[78].

In this context, AEA takes part in regulating sperm capacitation, by producing an increase in sperm calcium concentration via TRPV1 channels [71], [74]. The consistent absence of detectable TRPV1 activity in infertile sperm, concomitant with the low levels of AEA detected in seminal plasma of infertile men could lead to a reduced fertilizing capacity of AEA. In addition, as TRPV1 ion channels contribute to the choice between cell survival and death during spermatogenesis in murine sperm [49], a decrease of sperm TRPV1 could be at least partly responsible for the oligospermia of infertile men. In addition, AEA present in both seminal plasma and uterine fluids prevents premature capacitation in freshly ejaculated sperm via a CB_1_-dependent signalling pathway [10], [53], a defense mechanism that may be impaired in infertile men.

The ECS plays a physiological role in maintaining a quiescent, uncapacitated condition before sperm interacts with the oocyte [47]. Therefore, it may be speculated that the reduction of AEA causes infertile sperm to lose their quiescent state and with that, the ability to prevent premature capacitation. This could then precipitate a premature acrosome reaction rendering that sperm infertile by its inability to penetrate an oocyte *in vivo*, or indeed in assisted conception such as in *in vitro* fertilization. This hypothesis is further supported by work from one of our groups [79], where the converse occurred: direct exposure of sperm to recreational concentrations of THC reduced acrosome reactions *in vitro.*

Using an animal model, we have also shown how the deregulation of the endocannabinoid system markedly impaired spermatogenesis with reductions in total sperm count, depleted spermatogenic efficiency and impaired sperm motility by short and long term exposure to HU210, a selective agonist for CB_1_ and CB_2_ receptors [3].

Additionally, the present findings show for the first time that components related to 2-AG metabolism are present in human sperm, extending recent data in murine sperm [50]. Much alike AEA, we report an increased synthesis: degradation ratio of 2-AG in infertile *versus* fertile sperm, paralleled by a lower concentration of 2-AG in seminal plasma of infertile *versus* fertile men. Interestingly, a regulatory role of 2-AG has been identified at the start up of mouse epididymal sperm. In particular, along the epididymis, sperm from caput to cauda encounter a decreasing concentration of 2-AG, that induces them to acquire the potential to become motile through CB_1_ activation [80]. Such a 2-AG gradient is controlled by a tight equilibrium between DAGL and MAGL activity in the epididymal tissues [80]. In addition AEA and 2-AG, by acting extracellularly at CB_1_ (and AEA also intracellularly at TRPV1), may play a key-role in controlling the spatio-temporal interaction of sperm with oocyte and sperm–oocyte fusion [50]. Therefore, in infertile men a decrease of 2-AG levels in seminal plasma could also reduce the fertilizing capacity of sperm through a mechanism yet to be explored.

Failed fertilization occurs in up to 10% of *in vitro* fertilization treatment cycles. Since the majority of fertility treatments are self-funded, this is a major expense to infertile couples. The current evaluation of the fertility potential of the male partner, and hence the fertility treatment choice, is based on semen analysis. However, semen parameters have failed to discriminate fertile and sub-fertile men as seen in this study and also reported by Giwercman and colleagues [81]. The present identification of the ECS as a family of new biomarkers to determine male infertility with more accuracy has enormous potential in the fertility clinic.

In conclusion, we report for the first time the presence of ECS components of 2-AG-related metabolism in human sperm and we show an overall reduction of AEA and 2-AG biosynthesis in sperm from infertile *versus* fertile men. More interestingly, these findings suggest that the functional loss of TRPV1 in infertile sperm could cause a loss of capacitation including the acrosome reaction, thus affecting negatively the interaction between sperm and oocyte, and ultimately resulting in fertilization failure. This is the first characterisation of ECS in human fertile *versus* infertile sperm, and provides compelling data that identify a previously unknown defect in male fertility. Our results open the opportunity for therapeutic exploitation of ECS-targeted drugs to treat male fertility problems, as well as for exploiting differences in semen ECS constituents to diagnose infertility. The possible involvement of new players of endocannabinoid signalling, such as GPR55, remains to be addressed in an independent investigation.
